# Improving Sensory Differentiation: Refining the ‘Fruitiness’ Descriptor in Extra Virgin Olive Oil

**DOI:** 10.3390/foods14081390

**Published:** 2025-04-17

**Authors:** Ángel García-Pizarro, Agustí Romero, Daniel Schorn-García, Jokin Ezenarro, Montserrat Mestres, Laura Aceña

**Affiliations:** 1 Universitat Rovira i Virgili (URV), Department of Analytical Chemistry and Organic Chemistry, Chemosens Research Group, Campus Sescelades, Edifici N4, C/Marcel⋅lí Domingo 1, 43007 Tarragona, Spain; angel.garcia@urv.cat (Á.G.-P.); daniel.schorn@urv.cat (D.S.-G.); jokin.ezenarro@urv.cat (J.E.); laura.acena@urv.cat (L.A.); 2 Fruit Production Program, IRTA Mas Bové, Ctra. Reus-El Morell Km. 3.8, Constantí, 43120 Tarragona, Spain; agusti.romero@irta.cat

**Keywords:** extra virgin olive oil, fruitiness attribute, green fruity, multivariate analysis, ripe fruity, sensory analysis

## Abstract

Sensory analysis is a fundamental tool in evaluating extra virgin olive oil (EVOO) quality, playing an essential role in both consumer markets and international competitions that recognize and promote high-quality olive oils. Among the key attributes assessed, the fruitiness descriptor—subcategorized as green or ripe—is particularly significant, especially considering that higher green fruitiness is often associated with greater prestige. However, a clear methodological approach to distinguish between green fruitiness and ripe fruitiness perceptions, particularly in their overlapping zone, is still lacking. This study aims to establish precise criteria for defining these boundaries by analyzing monovarietal EVOOs produced from nine olive varieties at three maturity stages over two consecutive harvest seasons (2021/2022 and 2022/2023). Sensory assessments were conducted by the Official Tasting Panel of Virgin Olive Oils of Catalunya, ensuring representativeness across the different fruitiness perceptions. Volatile compounds of the samples were extracted using headspace solid-phase microextraction (HS/SPME) and separated and identified via gas chromatography–mass spectrometry (GC/MS). Multivariate analysis revealed three distinct volatile profiles corresponding to different sensory perceptions. These findings suggest that incorporating an intermediate sensory attribute between green fruitiness and ripe fruitiness could improve classification accuracy in both competitions and premium markets, enhancing the appreciation and valuation of high-quality EVOOs.

## 1. Introduction

Nowadays, a key objective of olive mills is to produce extra virgin olive oils (EVOOs) of the highest quality, with distinct properties that allow them to be distinguished from the much more uniform bulk productions. This differentiation enables producers to offer exclusive products to consumers. In this way, EVOOs with unique profiles—linked to specific regions, production methods or olive varieties—can then be presented at the numerous regional, national and international competitions held worldwide to recognize the best olive oils [1]. This recognition not only benefits producers but also has a significant impact on the broader economy of the region involved. By stimulating consumer interest in olive oil, it supports rural development through job creation in the olive-growing sector [2]. Furthermore, it promotes economic sustainability for producers who can market their oils as unique, high-quality products, enabling them to compete in premium markets with higher profit margins. This demand stimulates innovation within the olive oil sector to achieve higher standards in both quality and production efficiency. In this context, extra virgin olive oil represents a key sector of the agri-food industry, contributing significantly to the economic development of the major producer countries, such as Spain, Italy and Greece [3].

Virgin olive oil (VOO) is one of the few foods whose final quality classification—extra virgin (EVOO), virgin (VOO), ordinary virgin (OVOO) and lampante (LOO) olive oils—depends on a sensory assessment [4]. This sensory analysis must be performed by official tasting panels certified in descriptive analysis of EVOOs, according to the International Olive Council (IOC) T. 20 Doc. nº 15 [5], which requires a minimum of eight trained tasters. These tasters evaluate the absence (for EVOO) or the weak presence (for VOO) of organoleptic defects, as well as the intensity of three positive official attributes—fruitiness, bitterness and pungency—on a scale from 0 to 10. In certain cases, such as the Protected Designation of Origin (PDO) VOOs, additional specific sensory descriptors have to be evaluated according to the IOC method T. 20 Doc. nº 22 [6]. However, these standard classifications may not fully describe the organoleptic characteristics of an olive oil, even though the positive attributes are subcategorized based on the median scores assigned by official tasting panel members, resulting in subclassifications of delicate, medium or robust, and even though fruitiness can be further subcategorized as ‘green’ or ‘ripe’ if a significant number of panelists detect one of these specific attributes.

On the contrary, EVOO competitions often operate under different criteria, leading to subjective and inconsistent evaluations when determining which oils achieve recognition. Given the great impact that these evaluations and awards have on the olive oil market, establishing clearer distinctions among attributes becomes essential. Standardized parameters would assist tasting panels, thereby enhancing the reliability of evaluations and improving the classification of EVOOs.

Since the chemical composition of EVOO determines its organoleptic profile, its analysis is the most objective tool for characterizing this product. This composition comprises numerous compounds with diverse chemical structures, which are present in a wide range of concentrations and result from different sources [7,8]. Some factors influencing this composition, such as the olive cultivars, climatic conditions or geographical environment, cannot be modified. However, other factors, including agronomic practices or the production process, can be adjusted. Thus, specific actions and strategies can be applied to slightly modify the composition, but firstly, it is indispensable to identify which compounds are related to the different attributes. Understanding the pathways or mechanisms that originate these compounds allows for the enhancement of those sensory attributes most valued by consumers or associated with the highest quality products.

Currently, regarding the aroma of EVOOs, the type of fruitiness—green or ripe—is one of the most important attributes considered in the international olive oil competitions to determine the quality: the higher the green fruitiness, the more prestigious the EVOO. However, this attribute is challenging to assess, especially since current legislation allows for different optional organoleptic terms on labels based on its intensity and perception. Moreover, the lack of well-defined boundaries between these descriptors creates challenges for both producers and competitions. As a result, an olive oil may be classified as fruity when neither green nor ripe fruitiness predominates, as green fruity when it evokes green fruit or as ripe fruity when it is reminiscent of ripe fruit [4]. Identifying the factors responsible for this attribute in a sample is complex because, as previously explained, variables such as the olive variety, environmental conditions, agricultural practices, processing techniques and the ripening degree of olives significantly affect the olive oil composition and, consequently, its flavor [7,9,10]. It might be assumed that low maturity indices would guarantee high scores for the ‘green fruity’ attribute [11]. In fact, some producing regions have advanced the harvest dates to try to enhance this attribute; however, this strategy has not always yielded the desired results because some of the EVOOs obtained from earlier harvests were described as ripe fruity or even showed higher ripe fruitiness scores than green fruity EVOOs. Therefore, further investigation needs to be conducted.

The relationship between the different attributes and the chemical volatile composition of extra virgin olive oils has been studied previously, but to date, most studies have either provided broad reviews of all compounds or have specifically focused on identifying those responsible for sensory defects [7,9,12,13,14,15], leaving the compounds contributing to positive attributes less examined. Regarding the ‘fruity’ attribute, it is known that there is a series of C5 and C6 compounds directly related to it [16]; however, the individual contribution of each volatile compound to the green or ripe notes remains unclear. Furthermore, the boundary between these two perceptions has not yet been established because studies regarding this topic mainly focus on samples with either ‘green fruity’ or ‘ripe fruity’ description, assessed by an official tasting panel [17]. Therefore, given the significant impact of fruitiness on the quality and classification of EVOO, it is necessary to study the specific compounds and processes that affect this attribute. Moreover, this research should consider that, while odorant compounds are responsible for the aroma, their interaction with other volatile compounds can alter the sensory perception. Consequently, studying the entire volatile fraction could provide very useful information.

Therefore, the aim of this study is to develop a precise methodology for defining the boundaries between green fruitiness and ripe fruitiness within the fruitiness descriptor of olive oils, with a particular focus on the confluence zone where these perceptions overlap. To obtain well-differentiated EVOO samples that cover the full spectrum of the ‘fruity’ attribute, nine different olive varieties were harvested at three different maturity stages throughout the campaign for two consecutive campaigns: 2021/2022 and 2022/2023. All the monovarietal EVOOs obtained were sensorily analyzed by the Official Tasting Panel of Virgin Olive Oils of Catalunya, and each of them was positively assessed for at least one of the descriptors—‘green fruity’, ‘ripe fruity’, ‘green’ and ‘other ripe fruits’—in order to ensure the presence of the different perceptions of fruitiness. The solid-phase microextraction technique (SPME) was applied to the headspace of the samples (HS) to extract and preconcentrate the volatile compounds, which were then separated and identified by means of gas chromatography–mass spectrometry detection (GC/MSD). The correlation between the sensory descriptors assessed by the tasting panel and the volatile compounds chromatographically detected was finally established by applying multivariate analysis.

## 2. Materials and Methods

### 2.1. Samples

The experiment was carried out over two consecutive campaigns (2021/2022 and 2022/2023) at the Institute of Agrifood Research and Technology (IRTA)-Mas Bové experimental field (Constantí, Spain), which has a germplasm bank of olive varieties replicated from the official germplasm bank of Córdoba (Spain).

Seven olive varieties were selected: ‘Arbequina’, ‘Picual’, ‘Corbella’, ‘Empeltre’ and ‘Morrut’ from Spain and ‘Koroneiki’ and ‘Coratina’, representative of the Mediterranean area [18]. The same varieties and trees were harvested in each campaign at three different maturity stages, with olives collected from each tree every 21 days starting in October. To obtain samples at the overripe maturity stage, EVOO samples of ‘Arbequina’, ‘Picual’, ‘Leccino’ and ‘Verdiell’ were also collected during the campaigns of 2020/2021 and 2021/2022 from late January to late March.

In all cases, the harvest was performed manually with the help of a spiked scraper. Each time, around 5–7 kg of olives was harvested through an exhaustive procedure to ensure sampling from all parts of the tree. Two or three trees were sampled each time depending on the availability of varieties in the germplasm bank and the conditions of each campaign. Immediately after harvesting, the olives were cleaned, and leaves were manually removed. Then, olives were stored in fruit boxes until arrival at the pilot plant. For each olive box, the maturity index was calculated according to the method proposed by Uceda and Frías [19].

Information regarding the varieties, sampling dates and maturity indices of the samples is presented in Table 1. It should be noted that it was not possible to obtain olives at the highest maturity stage for some varieties due to the severe drought.

### 2.2. Sample Preparation

A total of 45 virgin olive oils were extracted using the ABENCOR^®^ system (ABENGOA, Seville, Spain) working under standardized conditions to minimize variability during the elaboration process and facilitate comparisons between varieties. Specifically, the olives were processed as soon as they arrived at the pilot plant of IRTA-Mas Bové after verifying that all fruits had an optimal sanitary status. The fruits were crushed in a hammer mill equipped with a sieve of 6 mm, and the conditions for the malaxation were 24 °C for 30 min. The resulting olive paste was then centrifugated to separate and collect the liquid fraction from the pomace. The liquid phase was transferred to a decanting funnel to remove excess water. Thereafter, the extracted oil was subjected to a second stage of high-speed centrifugation using a KUBOTA 8100 centrifuge (KUBOTA, Osaka, Japan). Finally, olive oil samples were filtered through a cellulose filter and transferred into bottles for their chemical and sensory assessment. Aliquots for the chromatographic analysis of the volatile fraction from each replicate were placed in separate vials and immediately stored at −18 °C until analysis.

### 2.3. Chemical Analysis

All samples were chemically analyzed by the Official Laboratory of the Catalan Government [20] to verify compliance with the legal limits for classification as extra virgin olive oil. Accordingly, acidity, peroxide index and UV-specific extinction coefficients K232 and K270 were determined, applying the official methods of analysis [21].

### 2.4. Sensory Analysis

The sensory analysis of all samples was performed by the Official Tasting Panel of Virgin Olive Oils of Catalunya, certified under ISO 17025 since 2002 and accredited by the Spanish National Accreditation Body (ENAC) for descriptive analysis of EVOO. The panel is officially recognized by the International Olive Council (IOC) and the European Union (EU). The sensory assessment was carried out according to the method established by the IOC [5], which requires a minimum of 8 official trained tasters to confirm the absence of organoleptic defects and to quantify the intensity of fruitiness, bitterness and pungency on a scale from 0 to 10 for classification as extra virgin olive oil. To ensure greater reliability, each sample was evaluated by at least 12 trained tasters in two separate tasting sessions.

After being classified as extra virgin olive oil, samples were then subjected to another descriptive sensory analysis according to IOC guidelines to evaluate additional specific sensory descriptors [6]. In this second session, tasters categorically indicate the presence or absence of ‘green fruitiness’, ‘ripe fruitiness’ or both. Moreover, the descriptors ‘green’ and ‘other ripe fruits’ were quantified on a continuous scale of intensity from 0 to 10 to support the classification of the fruitiness type: green or ripe. Furthermore, tasters also quantified other established sensory descriptors (e.g., sweet, almond, walnut, apple, etc.) and could freely propose new secondary attributes. However, only those secondary descriptors perceived by at least 33% of the judges were accepted and considered for analysis in this study [8].

In addition, the global sensory score algorithm developed by Romero Aroca [22] was applied by the panel. This algorithm, ranging from 0 to 9, supports the classification of virgin olive oils, requiring a minimum score of 6.5 to be qualified as extra virgin category.

### 2.5. Volatile Compound Analysis

The analysis of the volatile fraction of each sample was carried out immediately after the end of each campaign to prevent uncontrolled degradation of the EVOOs. The solid-phase microextraction (SPME) technique was applied to the headspace (HS) of the samples to extract and preconcentrate the volatile compounds in a single step, which were then separated and identified using gas chromatography coupled to mass spectrometry detection (GC/MSD). Each EVOO sample was analyzed in duplicate.

For this purpose, an adaptation of the method developed by Aparicio-Ruiz et al. [23] was followed. Specifically, 5.0 g of each EVOO was placed in a 20 mL glass vial together with a magnetic stirring bar and 0.2 g of 4-methyl-2-pentanol (purity above 98%) acting as internal standard (IS) at a concentration of 10 mg/kg. After tightly sealing the vial with a silicone septum under nitrogen atmosphere, the sample was tempered for 5 min at 35 °C in a thermostatic bath under constant magnetic stirring. Afterward, the stainless-steel needle of the SPME device was manually pushed through the vial septum, exposing a 2 cm DVB/CAR/PDMS (50/30 µm) fiber (SUPELCO, Merck KGaA, Darmstadt, Germany) to the headspace of the sample for 1 h. Then, the fiber was removed from the vial and inserted in the GC injection port for thermal desorption of the analytes at 270 °C for 1 min in the splitless mode.

The chromatographic analysis was performed with a Hewlett-Packard 6890 gas chromatograph (HP, Palo Alto, Santa Clara, CA, USA) equipped with an HP-5973 mass selective detector (HP, Palo Alto, CA, USA). A Chrompack CP-WAX 57CB fused silica capillary column (50 m × 0.25 mm i.d., 0.2 µm film thickness) from Agilent Technologies (Santa Clara, CA, USA) was used for separations, with helium as the carrier gas at a constant flow rate of 0.9 mL min⁻^1^ and a head pressure of 14.8 psi. The oven temperature program was 40 °C (5 min), 3.5 °C min^−1^ to 120 °C and 5 °C min^−1^ to 210 °C (1 min). The mass spectrometer operated in the electron impact ionization mode at 70 eV, and the interface, ion source and mass quadrupole temperatures were 200 °C, 230 °C and 150 °C, respectively. The mass-to-charge (*m*/*z*) ratio range was from 35 to 300 atomic mass unit (amu), and data were registered using the Mass Hunter software (version 10.0.368) from Agilent Technologies (Santa Clara, CA, USA).

### 2.6. Volatile Compound Identification

The identification of volatile compounds was performed by comparing the experimentally obtained mass spectra with those of pure standards from the NIST 17 library. In addition, retention indices (RIs) were calculated and verified to support the identification of each compound. The RI were determined from the retention times of a series of n-alkanes standards (C8–C20) (Merck KGaA, Darmstadt, Germany) prepared in hexane (~40 mg L^−1^ each) analyzed under the same chromatographic conditions.

The identification of the odor compounds within the volatile fraction was performed by consulting various odor databases [24,25,26,27].

### 2.7. Volatile Compound Relative Quantification

The peak area integration of each identified volatile compound was carried out using targeted conventional integration in Agilent Technologies Mass Hunter Qualitative Analysis 10.0 (Santa Clara, CA, USA). After integration, the relative areas (A′) were calculated by dividing the area of the volatile compound peak (VC) by that of the internal standard peak (IS), both normalized to the sample weight and the added IS, respectively, according to the following equation:

(1) $$A^{\prime }=\frac{\frac{VC\;area}{Sample\;weight}}{\frac{IS\;area}{IS\;weight}}$$

### 2.8. Statistical Analysis

Principal component analysis (PCA) was applied to the relative areas of the identified volatile compounds and used as an exploratory analysis to study the variability among data corresponding to different maturity stages and olive varieties. Moreover, the visualization of the PCA was also used to identify potential trends based on these variables and to detect outliers and remove them if necessary.

Partial least squares discriminant analysis (PLS-DA) was used to discriminate between the predefined maturity groups and the relative areas of the identified volatile compounds. Variables with high importance in prediction (VIP) were obtained from PLS-DA to be explored more thoroughly.

All data analyses were conducted using MATLAB R2022b (The MathWorks, Natick, MA, USA) and PLS Toolbox v9.2 (Eigenvector Research Inc., Eaglerock, CA, USA).

## 3. Results and Discussion

### 3.1. Exploratory Analysis

All olive oil samples obtained using the ABENCOR^®^ system were evaluated to establish their key chemical quality parameters before carrying out the sensory and volatile analyses. This includes the determination of free acidity, peroxide value and UV-specific extinction coefficients (K232 and K270), applying the official methods established by the International Olive Council and the EU Regulation. All 45 samples met the legal requirements to be classified as EVOOs.

Subsequently, headspace solid-phase microextraction (HS/SPME) followed by gas chromatography–mass spectrometry (GC/MSD) were performed in duplicate, enabling the identification and relative quantification of 153 compounds belonging to different chemical families, including ketones, aldehydes, esters, alcohols, acids, terpenes, ethers, sulfur compounds, nitrogenous compounds and hydrocarbons. In principle, only odor-active volatiles contribute to the aroma, although the synergistic and antagonistic effects exhibited by some volatiles should also be considered.

**Figure 1:**
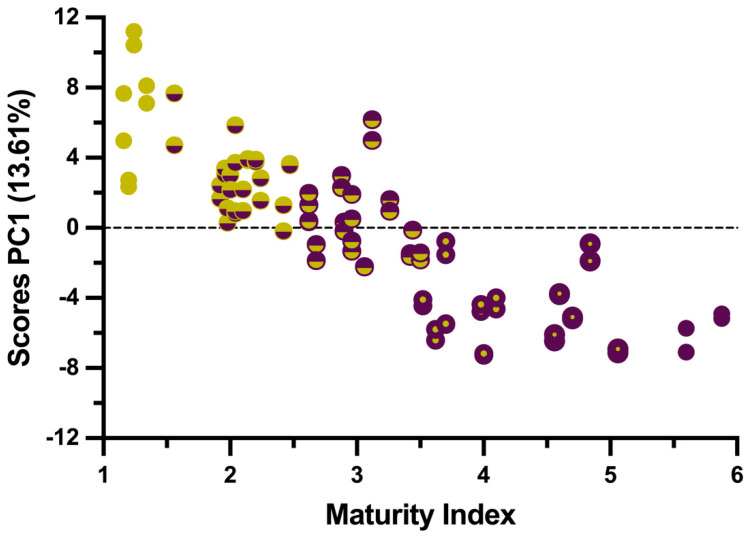
Score plot of PCA for the 45 EVOO samples analyzed in duplicate based on their identified volatile compounds and maturity indices (MIs). Colors are assigned to the scores according to their MI values, ranging from yellow (lowest MI) to purple (highest MI).

To better interpret these results in the context of aroma development, it becomes essential to assess the relationship between the identified compounds and the degree of maturation, as the volatile profile of EVOOs is influenced by fruit ripeness. Accordingly, the maturity index (MI) of the sampled olives was determined before they were processed to obtain the different EVOOs. This parameter, which ranges from 0 to 7, reflects the degree of olive fruit ripeness based on characteristic changes in skin and flesh color. At stage 0, both the skin and flesh are deep green, progressing to yellow skin at stage 1. At stage 2, less than half of the skin has turned purple, while at stage 3, more than half is dark. At stage 4, the skin is fully purple, but the flesh remains green. Stage 5 shows the first signs of purple flesh; stage 6 shows more than half of the flesh turning purple; and by stage 7, both the skin and flesh are completely purple. This color evolution reflects increasing ripeness and softening texture [19]. To explore intrinsic trends among the samples, principal component analysis (PCA) was applied to the dataset of identified volatile compounds and MI values. The resulting score plot (Figure 1) showed a clear distribution along PC1, with samples arranged from low to high MI values. This trend confirms that the studied EVOOs cover the entire range of maturity stages. Moreover, the close grouping of duplicates demonstrates the reproducibility of the volatile compound analysis.

The ripening pattern observed in the PCA scores allows us to establish three differentiated maturity ranges (MRs) to describe the progression of the olive maturation process. The first maturity range (MR1) represents early maturation and was set for MI values between 0.00 and 2.00, while the second maturity range (MR2) corresponds to an intermediate maturation stage and covers MI values from 2.01 to 3.50. The third maturity range (MR3) represents late maturation and was set between MI values 3.51 and 7.00. These classifications coincide with conventional thresholds used to determine the optimal harvesting period [10]. However, it is important to note that these MRs do not directly correspond to the predefined sampling dates of this study, as olive variety plays a key role in the maturation process [10,11]. As a result, certain varieties reach higher MI values earlier during the campaign, while others cannot reach the highest MI values due to genetic factors influencing ripening and drupe color development. This variability is reflected in Table 1, where the number of samples assigned to each MR differs despite the definition of three sampling dates.

### 3.2. Classification Analysis

**Figure 2:**
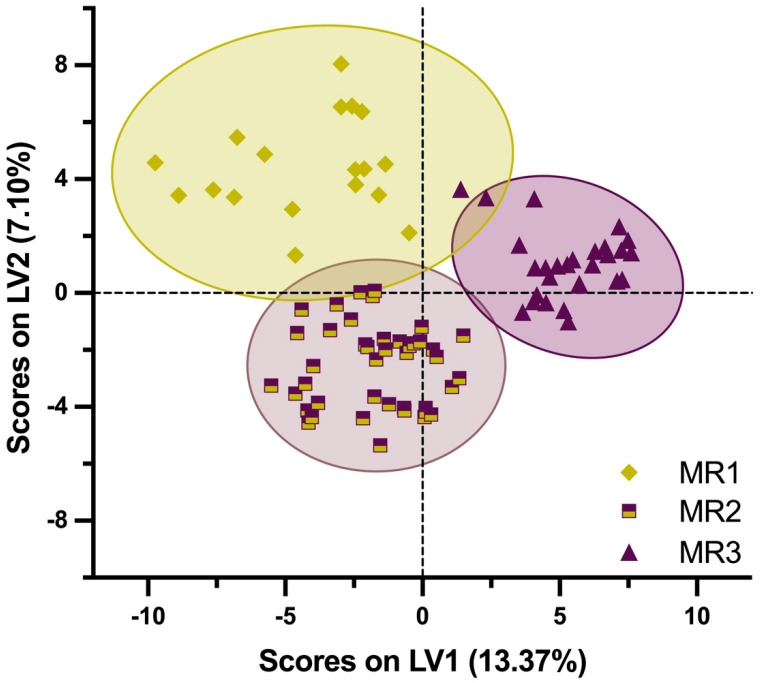
Score plot of PLS-DA for the 45 EVOO samples analyzed in duplicate regarding the relative quantification of all the identified volatile compounds and the maturity ranges (MRs) previously established and represented by different colors and shapes: yellow diamond for MR1; yellow/purple squares for MR2; purple triangles for MR3.

Following the successful differentiation of the different maturity stages of the EVOO samples using PCA, a supervised multivariate approach, partial least squares discriminant analysis (PLS-DA), was applied to further evaluate the extent to which the volatile profile effectively characterizes the three maturity ranges. Although it is known that different factors, from the olive variety to the olive oil extraction process, are responsible for the volatile fraction of EVOOs, it can be seen in Figure 2 that the volatile composition is modified by the phenological maturation process, represented by the maturity ranges considered.

The score confidence limits for each maturity range represented by colored ellipses in Figure 2 show a different variability for each maturity class throughout the ripening process. Although the sampling plan was designed to minimize the influence of different factors, such as the environmental conditions, the agricultural practices or the processing techniques, the inherent variability persists due to the biological differences among samples. The key factors contributing to this variation include the olive variety, harvest timing and specific ripening dynamics, which partially explain the overlap observed between the maturity ranges. The MR1 class, corresponding to the lowest maturity index (from 0.00 to 2.00), presents the highest variability, which can be attributed to the large number of olive varieties considered: Arbequina, Picual, Corbella, Empeltre, Morrut, Koroneiki and Coratina. Each one of these varieties initiates its ripening process at different times and follows a unique ripening evolution, even among trees of the same variety. As the maturation process progresses, ripening patterns become more homogeneous across both varieties and trees, resulting in lower variability for the MR2 and MR3 classes. By the final stage, olive drupes reach the highest maturity index (from 3.51 to 7.00), limiting further changes in this parameter and resulting in a lower variability for the MR3 class.

The PLS-DA model confirms a direct relationship between the volatile profile of the EVOO samples and the defined maturity ranges. Moreover, this analysis enables the identification and selection of the variables of importance in prediction (VIP), highlighting the most relevant volatile compounds that differentiate among maturity ranges with statistical significance. The selected VIP compounds, listed in Table 2 together with their relative areas, are associated with the maturity ranges they help distinguish.

### 3.3. Volatile Compounds

A total of 153 volatile compounds were identified in the analysis of all EVOO samples, although not all compounds were present in every sample. After compiling the relative areas for all samples, a combination of ANOVA and PLS-DA was applied to determine which compounds varied significantly among maturity ranges (MRs). ANOVA was used to identify those volatile compounds whose concentrations changed significantly throughout the ripening process, while PLS-DA facilitated the classification of samples by emphasizing the most relevant compounds in differentiating the maturity stages.

Despite the inherent variability in olive oil composition due to factors such as diverse cultivars, different ripening stages and climatic fluctuations in real orchard settings, the results in Table 2 show the identified volatile compounds that exhibit statistically significant differences in relative concentration between the three defined maturity ranges. Moreover, Table 2 specifies the compounds identified as variables of importance in projection (VIP) by the PLS-DA model for each maturity range. In general, but with some exceptions, VIP compounds associated with MR1 were those found at their highest concentrations in this early stage of ripening. Similarly, those linked to MR3 showed their higher concentrations at this most advanced maturity stage. However, most VIP compounds related to MR2 showed their lowest concentrations in the third stage. These findings suggest a transitional phase in the volatile profile between early and late ripening, with expected variations due to the complexity of volatile compound behavior, which will be further explored.

The volatile compounds identified include the major families typically found in virgin olive oils produced via the lipoxygenase pathway: acids, alcohols, aldehydes, esters and ketones [28,29]. In addition, hydrocarbons (both saturated and unsaturated), ethers and terpenes are present, together with nitrogenous and sulfur compounds, though in smaller numbers. Beyond these well-characterized groups, several other volatile compounds were identified, some of which have been less frequently reported in previous studies [30] or not widely studied [31]. Despite all samples being extracted from healthy fruits immediately after harvesting and under controlled conditions, small amounts of volatile compounds associated with degradative processes [32,33] were detected. Nevertheless, as will be discussed later, the sensory analysis confirmed that these compounds did not affect the overall quality of the samples.

The analysis of the obtained results shows a clear evolution in the volatile profile across maturity stages, supporting the expected transition from a green to a fruity aroma as ripening progresses. Specifically, the C5 alcohol family (1-penten-3-ol, (E)-2-pentenol, (Z)-2-pentenol) showed a decreasing trend from MR1 to MR3, with (Z)-2-pentenol presenting the highest concentration. Given their link to the fresh and herbaceous notes, their decrease coincides with the attenuation intensity of green attributes. Consequently, these compounds were identified as variables with high variable importance in projection (VIP) for MR1, suggesting they are the key markers in distinguishing early-maturity stages.

C6 alcohols, which contribute to both green and mildly fruity nuances, followed variable trends. On the one hand, 1-hexanol and (E)-2-hexenol increased from MR1 to MR2 before declining in MR3; on the other hand, (E)-3-hexenol and (Z)-3-hexenol remained nearly stable between MR1 and MR2 but decreased by more than 50% in MR3. These variations are related to enzymatic reactions occurring during maturation, as reported by Angerosa et al. [34], who attributed changes in C5 and C6 alcohols to alcohol dehydrogenase (ADH) activity in olive pulp and seeds. According to Table 2, these compounds were identified as important variables for differentiating between mid- and late-maturity stages, reflecting their contribution to the evolving aroma profile.

With regard to aldehydes, contrasting behaviors were observed. 2-propenal, 2-methylbutanal and 3-methylbutanal were found at concentrations below the limit of detection (LOD) in MR1 and MR2, but they increased significantly in MR3. This behavior coincides with the one observed by Inarejos-García et al. [35], who associated these compounds with a wet wood aroma. In contrast, 2-methyl-2-butenal reached similar high concentrations in MR1 and MR2, but it decreased below the LOD in MR3. Since this compound has been linked to ‘green fruity’ attributes [17], its drop further supports the transition away from fresh green notes in later maturity stages; therefore, these aldehydes were identified as important variables for characterizing the advanced ripening stage.

C6 aldehyde series, mainly hexanal and (E)-2-hexenal, are among the most abundant volatile compounds in EVOO and are essential for defining its sensory profile. Hexanal, associated with green and grassy notes, decreased significantly between MR2 and MR3. In contrast, (E)-2-hexenal—linked to green fruity notes—increased from MR1 to MR2 but decreased in MR3, suggesting a peak of this attribute at mid-ripening before evolving toward riper attributes. On the other hand, (Z)-2-hexenal (contributing to green notes), (E)-3-hexenal (associated with green and grassy aromas) and (Z)-3-hexenal (with a leaf-like aroma) progressively decreased throughout maturation. This overall reduction in green-aroma compounds highlights the decreasing fresh and green character of the olive oil as ripeness progresses. Accordingly, these aldehydes are identified as VIPs in distinguishing early- and mid-maturity stages.

The lipid oxidation product 4-oxo-2-hexenal, derived from ω-3 fatty acid degradation [36], also showed a progressive decrease in concentration along the three MRs. This trend coincides with the one found by Beltrán et al. [37], who observed a reduction in linolenic acid (ω-3) levels with the development of harvest, further reinforcing the biochemical basis of volatile changes.

Ester compounds, the key contributors to the fruity character of EVOO, showed trends consistent with ripening progression. Methyl 3-methyl-2-butenoate, associated with overripe fruit aromas in snake fruit [38], was not detected in MR1, but it reached its highest concentrations in MR3, confirming its role as a marker of advanced ripening. The other interesting esters were (Z)-3-hexenyl acetate, an ester contributing to green fruity notes that showed a decreasing trend, with significant differences only between MR1–MR2 and MR3; butyl acetate, linked to pear-like aroma, which was only detected in MR3, reinforcing the development of fruity notes in later stages. In contrast, hexyl acetate reached its highest level in MR2, potentially contributing to the balance of green and ripe attributes at this stage [29,39].

5-ethyl-2(5H)-furanone was found in higher concentrations in MR1, showing significant differences compared to MR2 and MR3. This compound, which provides a caramel-like aroma, is notably more abundant at the early stages of maturation. Similarly, γ-nonalactone, a compound providing a coconut-like fragrance, showed significant variation across all stages of maturation, with MR1 exhibiting higher concentrations compared to MR2 and MR3. Although these lactones were identified as VIPs for distinguishing the MR1 stage, their individual aromas were not perceptible at this point when carrying out the sensory analysis of the EVOO samples. This suggests that their role as VIPs may result from interactions with other volatile compounds, influencing the sensory characteristics of olive oil during early maturation.

Although terpenes are less abundant than alcohols and aldehydes, they play a crucial role in defining specific sensory attributes. Thus, for example, (Z)-β-ocimene reaches the highest concentration in MR2 before decreasing, which correlates with the floral descriptor detected in sensory analysis. Copaene, present in both MR1 and MR2, was undetectable in MR3. In contrast, α-cubebene—linked to mango-like notes—was most abundant in MR2. Interestingly, α-farnesene—known for its association with ripe fruit aromas—was most concentrated in MR3, further corroborating the fruity aromatic shift. Finally, it is remarkable that α-cubebene has been reported in higher concentrations in green fruity samples [17]. However, in this study, its highest levels coincided with MR2, a stage where green and fruity attributes are balanced, suggesting its role in defining an intermediate profile. This suggests that, although terpenes are often present below their odor thresholds, their contribution to the overall aroma should not be overlooked. Beyond their direct odor activity, terpenes may also influence perception through synergistic interactions with other volatile compounds, further shaping the overall sensory profile.

It is important to note that while the identification and trends of volatile compounds provide valuable insight into the evolution of the olive oil aromatic profile, these findings should be corroborated by sensory analysis to ensure their alignment with human perception. Sensory analysis remains crucial to confirm the sensory impact of these compounds, as the human olfactory system may interact with volatile compounds in complex ways that cannot be fully predicted from chemical data alone. As will be discussed in the next section, sensory results are essential for validating the volatile compounds’ contribution to the perceived aroma, allowing for a more comprehensive understanding of the sensory evolution of olive oils across maturity stages.

### 3.4. Sensory Analysis Results

According to the organoleptic assessment conducted by the Official Tasting Panel of Virgin Olive Oils of Catalunya, all of the studied virgin olive oils were classified as extra virgin, with global sensory scores exceeding 7, confirming their top quality. Since all samples were collected and processed under standardized conditions, this ensured that the differences observed were solely attributable to ripening effects rather than extraction process variables [40].

**Figure 3:**
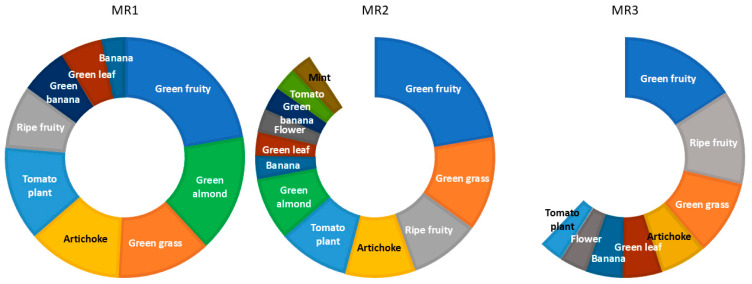
Solar projection of the median values of secondary attributes ordered from highest to lowest. The same color is used for the same attribute.

The fruitiness attribute of these EVOOs does not strictly show a ‘green’ or ‘ripe’ character but instead reflects a balanced combination of both, with varying intensities depending on the maturity stage. In addition to fruitiness, the sensory panel evaluated other secondary attributes, as described in Section 2.4. To ensure consistence and relevance, only those secondary descriptors perceived by at least 33% of the judges were considered. This selection is reflected in Figure 3, which presents a normalized projection of the medians of the evaluated attributes across the three maturity stages, offering a detailed composition of the descriptive sensory analysis for each maturity range (MR1, MR2 and MR3). Most of these secondary descriptors have been reported previously [8,41].

The sensory results are consistent with the classification analysis presented in Section 3.2 (PLS-DA model), reinforcing the observed trends. MR1 and MR2 have a similar dimension component of ‘green fruity’. In the case of MR2, however, this component begins to blend with the ripe component, altering the way tasters perceive the intensity of both green and ripe attributes. Finally, the MR3 sensory profile shows a distinctly ‘ripe fruity’ character, forming a third category, as previously observed in Figure 2.

In this context, these results also emphasize the importance of further training for tasters, as EVOOs retain their positive attributes up to an optimum maturity index of 3.5, corresponding to the end of the MR2 class in the present study. Beyond this stage, EVOOs should be classified into the ‘ripe fruity’ class, as their sensory profile is clearly poorer than that of earlier stages (Figure 3, MR3). However, in the current classification system, ‘ripe fruity’ is often used as soon as a fruit aroma is detected or when green or plant-like nuances are absent, which may not fully capture the sensory diversity of EVOOs. This broad classification may undervalue EVOOs from certain varieties, such as Arbequina, which naturally develop ‘fruity’ aromas even when derived from olives at an earlier maturity stage. As a result, these oils are often classified into the general ‘ripe fruity’ category despite potentially belonging to a distinct classification. This demonstrates the need to refine the classification criteria for the nuances of the official fruitiness attribute, as supported by our findings.

As shown in Figure 3, the sum of the medians for the low-maturity oils (MR1) is the highest among the three maturity ranges, indicating their greatest aromatic intensity. However, the diversity of sensory attributes in MR1 is lower than in mid-maturity oils (MR2) and comparable to high-maturity oils (MR3). Despite having a similar number of descriptors, high-maturity oils show a lower global aromatic intensity than MR1. This agrees with previous studies, which report that aromatic intensity decreases as ripening progresses [42,43].

‘Green fruity’ is the predominant attribute in all maturity ranges. However, in MR3, its overall value decreases compared to oils obtained from less mature olives. In contrast, the ‘ripe fruity’ attribute follows a different trend, increasing in prominence with maturation: it progresses from the sixth most prominent attribute in MR1 to the third in MR2 and becomes the second most prominent attribute in MR3.

The most prominent secondary attributes in MR1—after ‘green fruity’—are those associated with a green character: ‘green almond’, ‘green grass’, ‘artichoke’ and ‘tomato plant’, all of which appear at similar intensities, followed by ‘green banana’ and ‘green leaf’. These results corroborate the fact that the EVOOs in this group show a characteristic green sensory profile. For MR2, the overall aromatic intensities are similar to MR1, except for ‘green almond’, which becomes less dominant, while ‘ripe fruity’ gains importance. Given that ‘green almond’ is closely associated with specific C6 aldehyde compounds, its decrease at this stage may reflect changes in the volatile compound composition. In addition, ‘green grass’, ‘artichoke’ and ‘tomato plant’ become less important in intensity, while the ‘banana’ attribute rises, highlighting the fruity character of oils in the MR2 group. Furthermore, it is worth noting the emergence of ‘flower’ and ‘tomato’ attributes—both considered the markers of ripeness [44]—further supporting the progressive shift in sensory perception. These findings suggest that the consideration of ‘ripe’ should be reconsidered and potentially replaced with ‘fruity’ to better capture the sensory evolution of these oils. Finally, for MR3, and as previously noted, ‘green fruity’ continues to decrease in intensity, and ‘ripe fruity’ becomes notably the second most prominent attribute, with a marked increase in absolute intensity. It is worth mentioning that the ‘green grass’, ‘artichoke’ and ‘green leaf’ attributes gain relative prominence due to the absence of other key green attributes, such as ‘green almond’ and ‘green banana,’ which are no longer detected at this stage, or due to diminution of the ‘tomato plant’, which is relegated to the last position. Similar to the transition from MR1 to MR2, the ‘banana’ and ‘flower’ attributes continue to increase in MR3. At this stage, where aromatic intensity is significantly reduced due to the decrease in both ‘green’ and ‘fruity’ descriptors, the sensory profile aligns more closely with that of a ‘ripe’ EVOO.

## 4. Conclusions

This study established a methodology to delineate the boundaries between ‘green fruity’ and ‘ripe fruity’ descriptors of the official fruitiness attribute in extra virgin olive oils (EVOOs), focusing on the overlapping of these two sensory perceptions.

The application of solid-phase microextraction (SPME) to the headspace of samples, followed by gas chromatography–mass spectrometry detection (GC/MSD), allowed for the extraction, preconcentration and identification of volatile compounds. The subsequent multivariate analysis revealed significant correlations between specific volatile compounds and different stages of maturation, which also coincided with a differentiation between three stages by the sensory descriptors evaluated by the Official Tasting Panel of Virgin Olive Oils of Catalunya.

The study revealed significant changes in the concentrations of specific volatile compounds throughout the maturation process. Thus, in general terms, there was a progressive decline in C5 and C6 alcohols and aldehydes, while esters increased. These changes correspond with a reduction in green sensory notes and an enhancement of fruity and floral notes, reflecting the transition from green to ripe fruity profiles across the three evaluated stages of fruitiness.

Therefore, based on these results, the study further delineates EVOOs into three fruitiness categories:

- Green fruity EVOOs: Characterized by high aromatic intensity with predominant green sensory notes and elevated concentrations of related volatile compounds.
- Fruity EVOOs: Exhibit considerable aromatic intensity with a balance of green and fruity notes, indicating optimal maturity.
- Ripe fruity EVOOs: Show a significant decrease in aromatic intensity, with a limited range of aromatic notes and reduced concentrations of positive aromas.

Integrating comprehensive volatile profile analysis with sensory evaluations provides a scientific basis for enhancing the objectivity and precision of extra virgin olive oil (EVOO) categorization. This methodology, which refines the classification criteria, offers both producers and tasting panels a valuable tool to evaluate, differentiate and enhance the value of EVOOs based on their specific aromatic profiles. This approach enhances product differentiation and boosts consumer satisfaction.

## Figures and Tables

**Table 1:** Maturity ranges (MRs) according to the maturity indices (MIs) intervals calculated based on the method by Uceda and Frías [19] and the number of samples assigned to each MR.

Maturity Range	From MI	To MI	No. of Samples	Mean MI
MR1	0.00	2.00	9	1.60
MR2	2.01	3.50	21	2.72
MR3	3.51	7.00	15	4.55

**Table 2:** Results of ANOVA and Tukey’s test of volatile compounds statistically significant for differentiation between classes. Mean relative area (A′ (×10^3^)) of the peak area for each identified volatile compound and maturity range: MR1, MR2 and MR3.

RI ^1^	Volatile Compound	Odor Descriptor	A′ MR1	A′ MR2	A′ MR3	VIP ^2^ for
<800	Pentane	Odorless	5.5 ^a^	3.2 ^a^	21.7 ^b^	MR3
<800	1,4-pentadiene	Mild hydrocarbon	62.5 ^b^	59.0 ^b^	22.7 ^a^	MR2
<800	Heptane	Odorless	17.3 ^b^	4.4 ^a^	16.6 ^b^	-
<800	Dimethyl sulfide	Cabbage, sulfur	1.0 ^a^	2.6 ^a^	6.9 ^b^	MR3
<800	2-methyl-heptane	Gasoline-like	15.6 ^b^	7.0 ^ab^	1.2 ^a^	-
801	Octane	Odorless	14.6 ^ab^	14.5 ^a^	19.7 ^b^	-
816	Acetone	Solvent-like, sweet	15.2 ^a^	33.6 ^ab^	57.4 ^b^	-
823	Methyl acetate	Fruity, sweet	4.2 ^b^	1.4 ^a^	04.7 ^b^	-
837	2-propenal	Pungent, acrid	<LOD	<LOD	0.6	MR3
904	2-butanone	Sweet, acetone-like	2.4 ^ab^	0.7 ^a^	4.3 ^b^	MR3
912	2-methylbutanal	Malty	<LOD	<LOD	1.8	MR3
915	3-methylbutanal	Malty	<LOD	<LOD	1.1	MR3
937	Benzene	Sweet, solvent-like	1.6 ^b^	0.3 ^a^	2.8 ^b^	MR3
947	Ethanol	Sweet	36.7 ^b^	19.2 ^a^	24.3 ^ab^	MR1
956	3,4-diethyl-1,5-hexadiene (RS/SR)	Mild hydrocarbon-like	40.2 ^b^	38.4 ^b^	15.4 ^a^	MR2
962	3,4-diethyl-1,5-hexadiene (Meso)	Hydrocarbon-like	34.4 ^b^	32.1 ^b^	13.2 ^a^	MR1, MR2
978	3-pentanone	Fruity, sweet	15.4 ^a^	21.9 ^b^	15.3 ^a^	MR2
1000	Decane	Alkane, odorless	1.6 ^ab^	1.0 ^a^	2.6 ^b^	MR3
1007	(Z)-3-ethyl-1,5-octadiene	Hydrocarbon-like	174.1 ^b^	166.4 ^b^	67.5 ^a^	MR2
1020	1-penten-3-one	Green, pungent	290.6 ^b^	294.3 ^b^	135.3 ^a^	MR2
1035	Toluene	Paint	113.5 ^b^	50.3 ^a^	137.7 ^b^	MR3
1047	2-methyl-2-butenal	Green, pungent	1.5 ^a^	1.1 ^a^	<LOD	MR1
1061	α-fenchene	Camphor	0.2	<LOD	<LOD	MR1
1072	Butyl acetate	Fruity, pear	<LOD	<LOD	0.6	-
1074	(E,E)-3,7-decadiene	Green, waxy	51.8 ^b^	53.3 ^b^	17.1 ^a^	MR2
1081	Hexanal	Green, grass	326.9 ^ab^	345.4 ^b^	230.6 ^a^	MR2
1083	4,8-dimethyl-1,7-nonadiene	Herbal, green, balsamic	43.7 ^a^	35.5 ^a^	<LOD	-
1110	(Z)-2-pentenal	Green, pleasant	13.5 ^c^	9.6 ^b^	4.8 ^a^	MR1
1123	Ethylbenzene	Gasoline-like	8.1 ^b^	5.0 ^a^	16.4 ^b^	MR3
1128	p-xylene	Solvent-like	2.0 ^a^	0.9 ^a^	10.3 ^b^	MR3
1135	m-xylene	Plastic	<LOD	<LOD	18.3	MR3
1137	(Z)-3-hexenal	Leaf-like	184.4 ^b^	66.7 ^a^	56.0 ^a^	MR1
1142	(E)-3-hexenal	Green, grass	729.8 ^c^	186.5 ^b^	58.9 ^a^	MR1
1160	1-butanol	Medicine, mild sweet	<LOD	<LOD	00.4	MR3
1168	Methyl 3-methyl-2-butenoate	Overripe fruity, papaya	<LOD	01.1 ^a^	13.6 ^b^	MR3
1171	1-penten-3-ol	Butter, pungent	74.4 ^b^	67.1 ^b^	36.1 ^a^	MR1, MR2
1207	(Z)-2-hexenal	Green	314.6 ^c^	148.3 ^b^	59.1 ^a^	MR1
1226	(E)-2-hexenal	Green apple-like, bitter almond	2009.7 ^a^	3619.1 ^b^	1481.2 ^a^	MR2
1249	(Z)-β-ocimene	Citrus, herb, flower	0.5 ^a^	44.8 ^b^	27.9 ^b^	MR2
1259	3-octanone	Mushroom-like, earthy	<LOD	<LOD	00.4	MR3
1276	Hexyl acetate	Fruity, sweet	4.4 ^a^	12.2 ^b^	6.1 ^ab^	MR2
1282	1,2,4-trimethylbenzene	Plastic	2.8 ^b^	1.5 ^a^	3.1 ^b^	-
1298	Acetoin	Butter, cream	0.1 ^a^	0.3 ^a^	1.3 ^b^	-
1321	(Z)-3-hexenyl acetate	Green, apple	69.8 ^b^	74.4 ^b^	24.7 ^a^	MR2
1330	(E)-2-pentenol	Mushroom	8.9 ^b^	7.9 ^b^	3.3 ^a^	MR1, MR2
1339	(Z)-2-pentenol	Green, plastic, rubber	104.5 ^b^	91.4 ^b^	36.7 ^a^	MR1, MR2
1374	1-hexanol	Resin, flower, green	21.4 ^a^	56.3 ^b^	26.6 ^a^	MR2
1382	(Z)-3-hexenol	Leaf-like, grass	1.8 ^b^	2.4 ^b^	0.4 ^a^	MR2
1389	Methyl 3-hydroxy-3-methylbutanoate	Ester	<LOD	0.6 ^a^	5.3 ^b^	MR3
1391	2-methyl-2-cyclohexenone	Mild wood	1.4 ^b^	0.1 ^a^	<LOD	MR1
1404	(E)-3-hexenol	Moss, fresh	182.4 ^b^	188.1 ^b^	54.9 ^a^	MR2
1411	(Z,E)-2,4-hexadienal	Green	81.1 ^c^	42.4 ^b^	18.5 ^a^	MR1
1418	(E,E)-2,4-hexadienal	Green	186.1 ^c^	102.5 ^b^	43.7 ^a^	MR1
1427	(E)-2-hexenol	Herb, leaf, walnut	12.8 ^a^	57.1 ^b^	17.4 ^a^	MR1
1490	Copaene	Wood, spice	0.1 ^a^	0.7 ^a^	<LOD	MR2
1498	α-cubebene	Herb, wax, mango	0.8 ^a^	8.3 ^b^	0.5 ^a^	MR2
1510	2-ethyl-1-hexanol	Rose, green	0.6 ^a^	4.6 ^b^	0.6 ^a^	MR2
1537	2,4-dimethyl-2-hexene	Slight hydrocarbon	8.5 ^b^	1.8 ^a^	0.6 ^a^	-
1564	Propanoic acid	Pungent, rancid	0.5 ^a^	0.9 ^b^	1.4 ^b^	MR3
1565	Valeric anhydride	Rancid	7.5 ^b^	2.0 ^a^	0.1 ^a^	MR1
1592	2-methyl propanoic acid	Rancid, butter, cheese	0.4 ^a^	0.6 ^a^	3.1 ^b^	MR3
1616	4-oxo-2-hexenal	Metallic, green	46.0 ^c^	9.9 ^b^	2.3 ^a^	MR1
1662	Dimethyl sulfoxide	Garlic	0.9 ^a^	1.7 ^b^	0.7 ^a^	MR2
1738	α-muurolene	Wood	<LOD	0.4	<LOD	MR2
1753	α-farnesene	Wood, sweet	<LOD	0.9 ^a^	4.1 ^b^	MR3
1768	2-cyclohexen-1,4-dione	Slight sweet	5.3 ^c^	1.5 ^b^	0.4 ^a^	MR1
1787	Methoxy-phenyl-oxime	Flower	14.4 ^b^	27.0 ^c^	5.1 ^a^	MR2
1803	5-ethyl-2(5H)-furanone	Spice, caramel	28.9 ^c^	7.7 ^b^	2.3 ^a^	MR1
1815	2-methyl-2-butenoic acid	Spicy, rancid	0.9	<LOD	<LOD	MR1
1854	Anethole	Anise, fennel	0.5 ^a^	0.5 ^a^	2.2 ^b^	MR3
1961	2-phenylethanol	Honey, rose, lilac	6.3 ^b^	5.2 ^b^	1.6 ^a^	-
1996	3-hexenoic acid	Cheese, green, grassy	3.0 ^b^	0.8 ^a^	0.3 ^a^	MR1
>20,000	γ-nonalactone	Coconut, peach	55.5 ^c^	17.9 ^b^	1.8 ^a^	MR1

^1^ Retention indices (RIs) calculated from the retention times of a series of n-alkanes (C8–C20) standards prepared in hexane. ^2^ Variables of importance in projection (VIP). In the rows, means with the same letter are not significantly different according to Tukey’s test (*p* < 0.05).

## Data Availability

The data presented in this study are available on request from the corresponding author.
